# Next-generation compact antenna for robust defense and CubeSat communication

**DOI:** 10.1038/s41598-026-37874-4

**Published:** 2026-02-06

**Authors:** Swati Varun Yadav, Manish Varun Yadav, S. Raghavendra, Vikas Gupta

**Affiliations:** 1 Manipal Institute of Technology, Manipal Academy of Higher Education, Manipal, India; 2 Department of Electronics and Telecommunication Engineering, Vidyavardhini’s College of Engineering and Technology, Vasai-Virar, Maharashtra India

**Keywords:** Compact antenna, Wideband, Defense communication, CubeSat comm, SDG-9, SDG-11, High-frequency electronics, Antenna engineering, Next-generation wireless networks, Engineering, Physics

## Abstract

The article presents a miniaturized ultra-wideband (UWB) antenna tailored for modern defense and small satellite communication requirements. Designed and optimized using CST Microwave Studio, the antenna delivers superior electromagnetic performance across both Sub-6 GHz and millimeter-wave frequency ranges. Realized on an FR4 substrate with overall dimensions of 10 × 12 × 1.5 mm³, the prototype achieves an impressive operating bandwidth of 3.4–14 GHz, equivalent to an impedance bandwidth of 121.8%. The measured results highlight a peak gain of 4.56 dBi, a return loss of -28 dB, and a radiation efficiency of 82.9%, ensuring reliable performance over a broad spectrum. With an electrical size of 0.113λ × 0.136λ × 0.017λ, the proposed design demonstrates remarkable compactness while maintaining stable radiation patterns and high efficiency. These characteristics make the antenna a strong candidate for resilient, interference-resistant, and high-performance applications in defense systems and CubeSat missions.

## Introduction

The advancement of modern wireless and defense communication systems has created a strong need for compact, efficient, and wideband antennas. Critical applications such as radar, surveillance, secure data transfer, and CubeSat communication demand antennas that can function effectively across broad frequency ranges while remaining suitable for size-limited platforms. Designing such antennas requires achieving wide impedance bandwidth, high gain, and stable radiation performance within a compact structure. With the emergence of Sub-6 GHz, millimeter-wave, and 5G technologies, antenna designs must address the dual challenges of miniaturization and performance enhancement. Compact antennas capable of covering multiple frequency bands are increasingly important for ensuring reliability and versatility in next-generation wireless and defense applications. In this work, a compact ultra-wideband antenna is presented, fabricated on an FR4 substrate with dimensions of 10 × 12 × 1.5 mm³. The design covers the 3.4–14 GHz range, offering high efficiency, low return loss, and stable radiation characteristics. Its compact form factor and wide operating bandwidth make it a strong candidate for applications in S-band, C-band, X-band, 5G systems, and nanosatellite communication platforms.

## Related work

 Ongoing research in microstrip patch antenna engineering has produced a variety of compact architectures capable of delivering improved wideband performance and reliable radiation behavior. In particular, planar ultra-wideband antenna designs incorporating modified radiator geometries together with defected ground structures have demonstrated effective impedance bandwidth enhancement while maintaining a low-profile form factor suitable for integrated wireless platforms^1^. In parallel, the growing demand for high-frequency wireless systems has driven the development of wideband microstrip antennas operating in the vicinity of 28 GHz, especially for fifth generation (5G) communication applications^2^. Further studies have explored millimeter-wave microstrip antenna solutions tailored for 5G mobile networks, underscoring the importance of geometry-specific optimization to achieve efficient operation at elevated frequencies^3^. Moreover, broadband U-slot antennas with dual-beam characteristics have been proposed, offering controlled radiation behavior alongside wide operating bandwidths for advanced communication systems^4^.

Building upon these developments, the present work focuses on the analysis of symmetrically shaped antennas by evaluating their radiation characteristics, resonant frequencies, size constraints, and substrate materials. Techniques to extend bandwidth are also examined, drawing on concepts from both physics and circuit theory to enhance performance across diverse operating environments^5–8^. In the context of millimeter-wave and terahertz applications, addressed the inherent distance limitations at these high frequencies and provided guidance for optimizing communication in these bands^9^. Furthermore, discussed the opportunities and challenges of using the terahertz spectrum as a new frontier in wireless communications^10^. Overall, this study reviews the evolution of microstrip patch antennas and highlights their significance in present and future wireless communication systems. Recent years have witnessed extensive research on compact and ultra-wideband (UWB) antenna designs to meet the growing demands of modern wireless, satellite, and defense communication systems. Various monopole and microstrip-based configurations have been explored to enhance bandwidth while maintaining compact dimensions, such as curved disc-monopole structures and extended circular patch designs with partial ground planes^11,12^. Parasitic elements, slot loading, and variable ground-plane geometries have been shown to significantly improve impedance bandwidth and radiation stability, particularly for satellite and defense-oriented applications^13–15^. Several studies have also focused on broadband and miniaturized planar antennas employing semi-circular slots, defected ground structures, and modified radiator shapes to achieve wideband performance suitable for UWB systems^16–21^. More recent works have extended these concepts toward multi-band and 5G microwave applications, covering S-, C-, and X-bands with compact planar geometries^22–24^, while others have addressed MIMO integration and band-notch characteristics for interference mitigation^25,26^. Despite these advances, many existing designs still face trade-offs between bandwidth, electrical size, and radiation efficiency, motivating the present work, which aims to achieve a superior bandwidth-to-size ratio with stable radiation characteristics suitable for defense and CubeSat communication platforms. By consolidating recent advancements and innovative approaches, the aim is to support ongoing efforts to improve connectivity and enable next-generation communication technologies.

### Antenna geometry

**Fig. 1:**
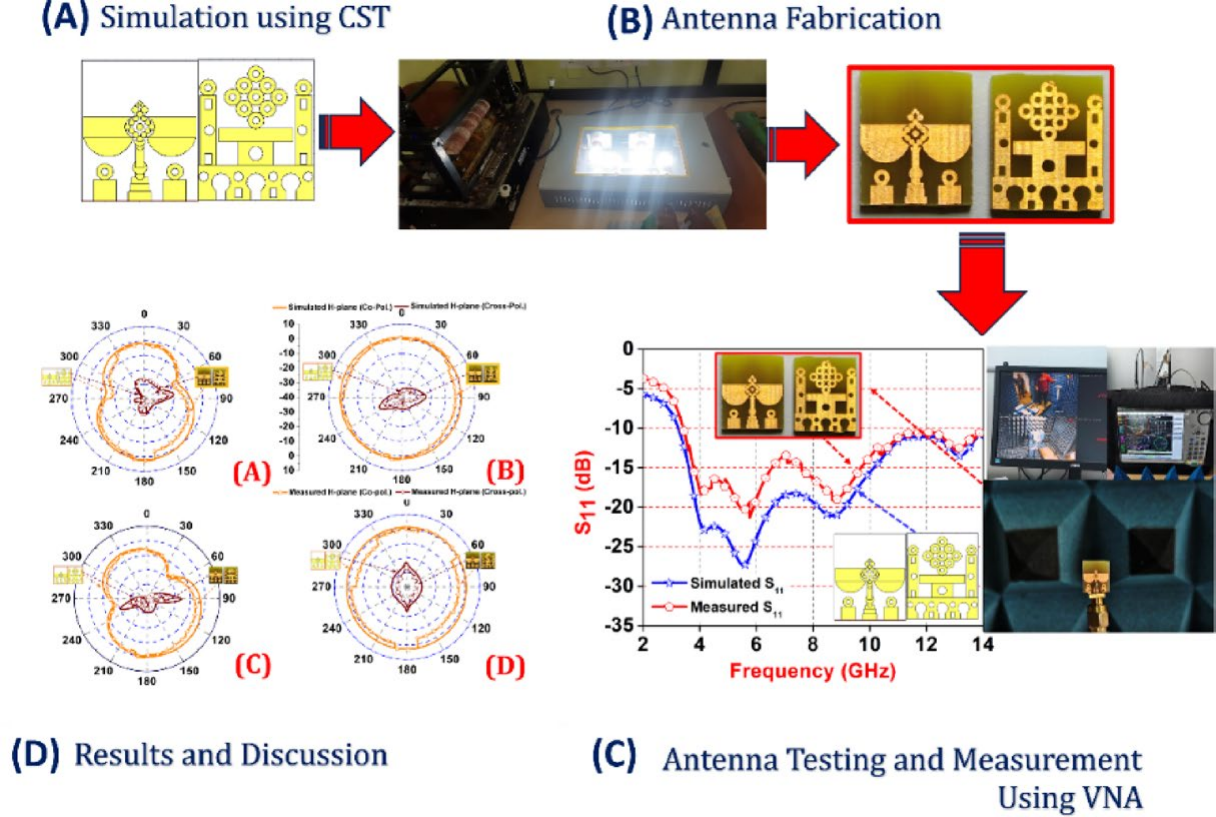
Step-by-step methodology for the proposed antenna design process. (**A**) Antenna geometry simulated using CST Microwave Studio; (**B**) Fabrication of the antenna prototype using standard PCB techniques; (**C**) Testing and measurement setup using a Vector Network Analyzer (VNA) in an anechoic chamber; (**D**) Comparison of simulated and measured return loss (S₁₁).

Figure 1 provides a comprehensive visual representation of the step-by-step methodology adopted for designing, fabricating, and validating a compact wideband antenna intended for millimeter-wave applications. The process begins with (A) Simulation using CST Microwave Studio, where the proposed antenna geometry is modelled and analysed. The design incorporates intricate patterns to achieve the desired bandwidth and gain characteristics. Using full-wave electromagnetic simulations, important performance metrics such as return loss (S₁₁), impedance bandwidth, and radiation patterns are evaluated.

These simulations help refine the design parameters before moving toward physical implementation. In (B) Antenna Fabrication, the optimized design is transferred onto an FR4 substrate using standard photolithography and etching techniques in a PCB fabrication lab. The fabricated prototype features both front and back views, showcasing the precise realization of the simulated layout. Care is taken during fabrication to maintain accuracy in dimensions, which is critical for high-frequency performance. (C) Antenna Testing and Measurement involves experimental validation of the antenna using a Vector Network Analyzer (VNA) inside an anechoic chamber. This setup ensures minimal external interference during the measurement of S-parameters and radiation characteristics. The fabricated antenna is connected to the VNA using an SMA connector, and its real-time behavior across the frequency band is recorded. The anechoic chamber, lined with RF-absorbing materials, enables accurate radiation pattern and gain measurements. Finally, (D) Results and Discussion compare the simulated and measured S₁₁ values across the target frequency range. The graph reveals a good agreement between the simulation and experimental data, with both showing a return loss below − 10 dB over a broad bandwidth, indicating efficient impedance matching. Minor deviations are attributed to fabrication tolerances, connector mismatches, and environmental conditions. Inset images in this section highlight key stages, including the fabricated antenna, simulation layout, and the test setup, supporting the validity of the design process.

**Fig. 2:**
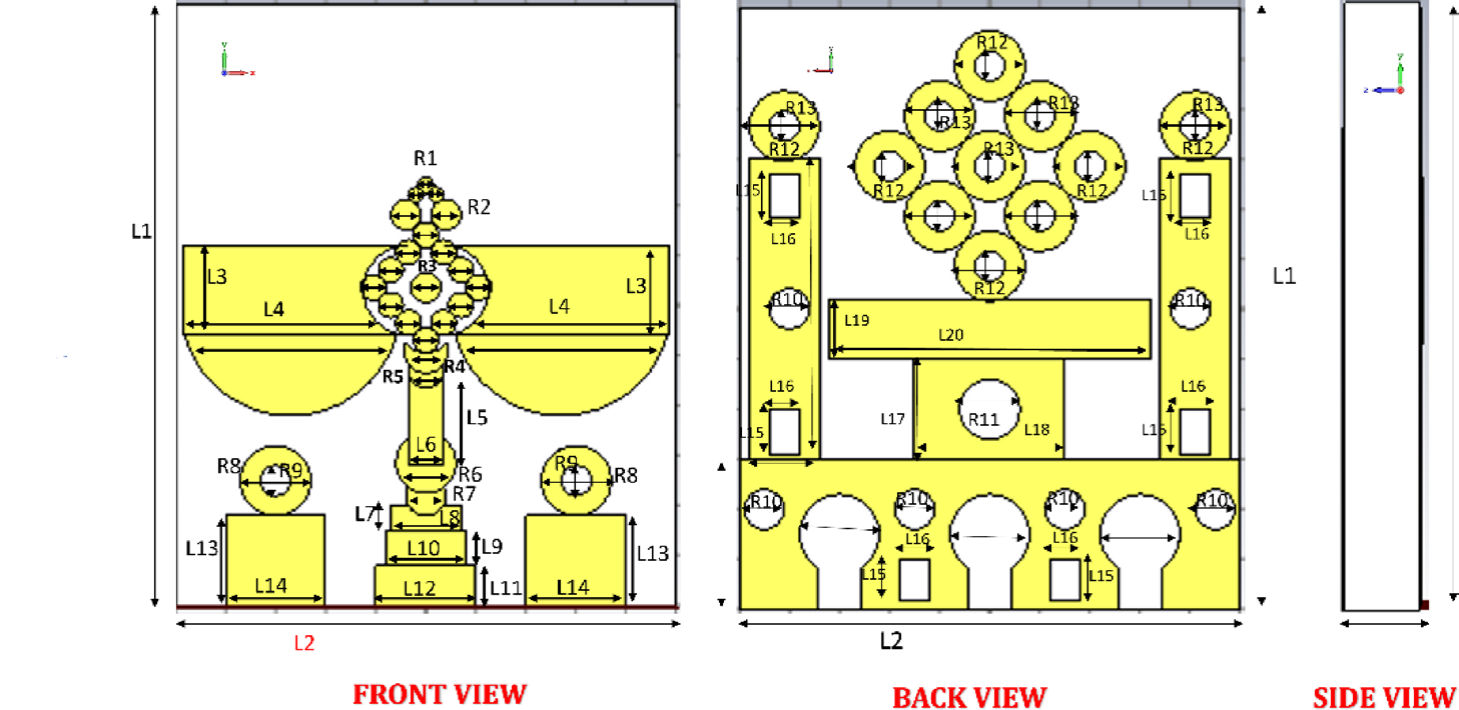
Graphical view summarizing of radiator.

**Table 1:** Proposed antenna Parameters values in (mm).

Parameters	L1	L2	L3	L4	L5	L6	R1	R2	L23
Values	12	10	3	0.2	1.5	0.3	1.4	0.9	6
Parameters	R8	R9	R10	R11	R12	L9	L10	R13	L24
Values	0.8	0.25	0.4	0.45	0.35	1.9	0.7	0.6	1.4
Parameters	R14	L17	L18	L19	L20	L21	L22	R15	L25
Values	0.4	1.5	1.9	2	0.7	1.6	0.8	0.7	0.6
Parameters	L29	L30	R17	L31	R18	L32	L33	R19	L26
Values	1	1	0.4	3	0.8	0.9	0.9	0.5	0.9
Parameters	R4	R5	L7	L8	R6	R7	R3	L34	L27
Values	0.3	0.75	1.8	4.5	2.2	0.15	0.4	1.5	0.9
Parameters	L12	L13	L14	L15	L16	L17	L11	L28	R16
Values	0.5	0.5	3	2.5	0.5	1.4	2.9	1.2	2.8

 Figure 2 presents the detailed dimensional layout and structural design of the proposed compact antenna through front, back, and side views, complemented by a tabulated list of physical parameters in millimeter as shown in Table 1. The overall dimensions of the antenna are defined as L_2_, L_1_, and L_21_ for its length, height, and width, respectively. The Front View illustrates the top layer, which features a unique radiating structure incorporating circular, rectangular, and semicircular elements precisely arranged for optimal electromagnetic performance. A distinctive diamond-shaped configuration is formed at the center using nine circular patches, each labelled as R_3_, symmetrically arranged around a central circular patch. This diamond structure is directly attached to the main microstrip feed and spans an area of L_12_ × L_11_, contributing significantly to the antenna’s resonance behavior.

To enhance performance in the lower frequency band, two rectangular patches of size L_4_ × L_3_ are integrated on either side of the diamond structure. Beneath these patches, a semicircular stub, referred to as R_14_, is connected to improve impedance matching. The Back View reveals the defected ground structure (DGS), which includes a rhombus-like element marked as R_12_ and R_13_, facilitating broad bandwidth and gain improvement. At the base of this structure, two rectangular slots with dimensions L_20_ × L_19_ and L_18_ × L_17_ are embedded, while two additional rectangular patches, L_22_ × L_23_, are placed laterally to support higher-order resonant modes. The Side View confirms the compact vertical profile, ideal for integration into miniaturized millimeter-wave systems. Together, these intricately defined structural elements ensure wideband performance, efficient radiation characteristics, and applicability in high-frequency, space-constrained environments such as 5G and defense communication systems.

### Evolution and parameter analysis of the antenna

**Fig. 3:**
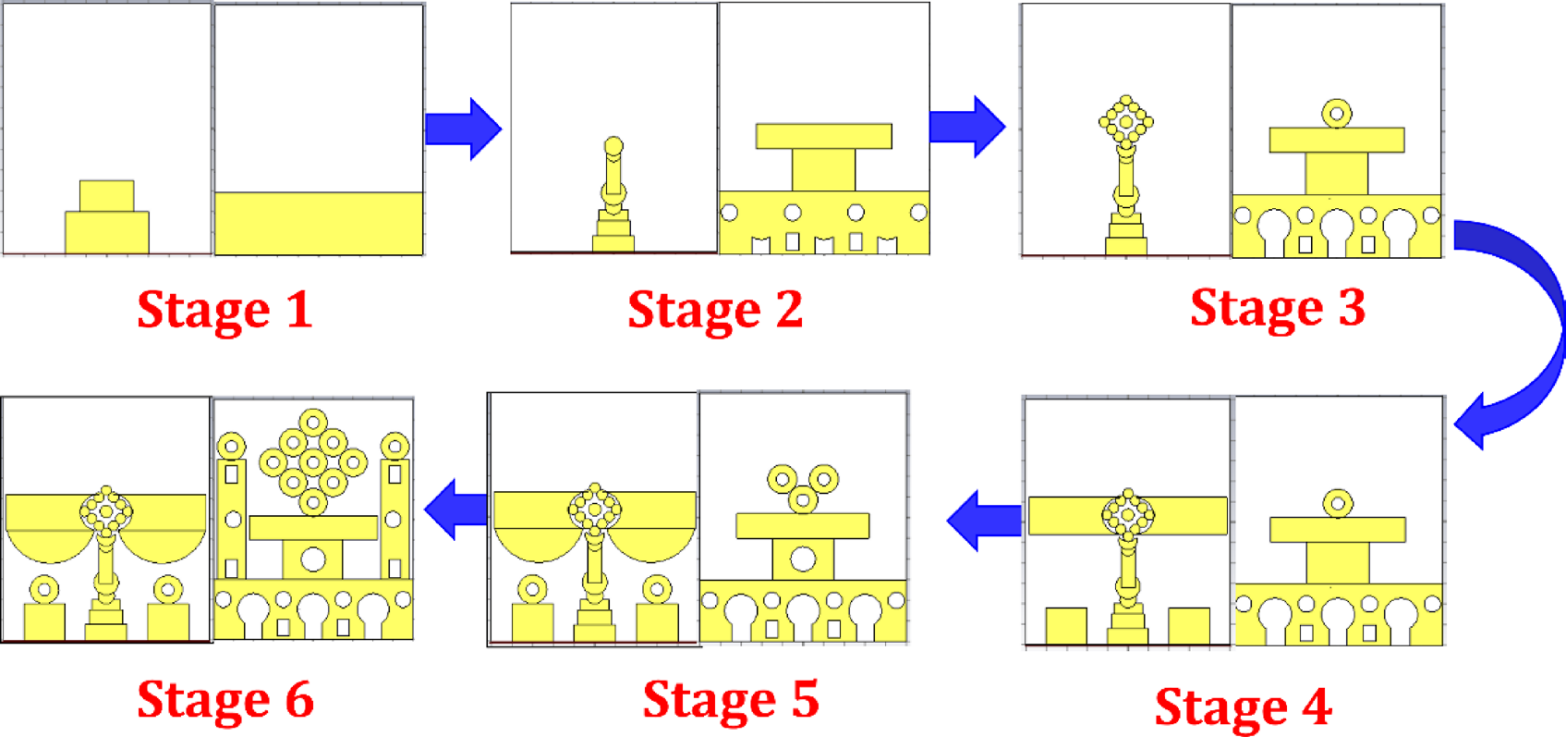
Making of the proposed antenna.

Figure 3 illustrates a 06 evolutionary design process for the proposed wideband antenna structure, capturing both front and back views at each development step. Each stage builds upon the previous one by progressively modifying the geometry to enhance performance of impedance bandwidth.

Stage 1 establishes the initial antenna configuration using a stepped rectangular patch on the top layer combined with a partially etched rectangular ground plane on the opposite side. This foundational structure defines the substrate characteristics, feeding arrangement, and baseline impedance behavior, serving as a reference geometry for subsequent design refinement.

In Stage 2, the antenna geometry is modified by introducing a vertically oriented cylindrical element on the radiator side, along with multiple circular and square slots in the ground plane. These changes initiate resonance tuning by modifying the effective electrical dimensions of the structure. The ground-plane alterations represent the early formation of a defected ground structure (DGS), which contributes to improved impedance matching over an extended frequency range.

Stage 3 further refines the design by incorporating a diamond-shaped arrangement of circular patches on the front layer, enhancing structural symmetry and increasing the number of resonant paths. In parallel, centrally positioned circular slots are introduced in the ground plane, supporting Multi-resonant behavior and marking the transition toward wider impedance bandwidth.

In Stage 4, large semicircular elements are symmetrically added adjacent to the central diamond structure, effectively increasing the electrical path length and improving impedance continuity across the band. Additional circular and rectangular slots are embedded in the ground plane to fine-tune the resonant modes. A circular ring placed above the central rectangular feature assists in adjusting higher-order resonances.

Stage 5 continues the geometric optimization by integrating paired semicircular patches along the sides of the main vertical structure and circular stubs near the lower edges of the radiator. These modifications help stabilize the impedance response over a broader frequency range. Simultaneously, the ground-plane layout is symmetrically refined to maintain consistent wideband impedance behavior.

Finally, Stage 6 represents the fully optimized antenna configuration, combining a nine-element diamond-shaped circular array, symmetric semicircular arms, and auxiliary circular stubs on the radiator side, along with a carefully engineered defected ground structure on the reverse. This final geometry achieves a continuous ultra-wide impedance bandwidth from 3.4 to 14 GHz, with quantitative radiation characteristics evaluated and reported exclusively for this optimized design, making it suitable for Sub-6 GHz and millimeter-wave wireless, defense, and CubeSat communication applications.

**Fig. 4:**
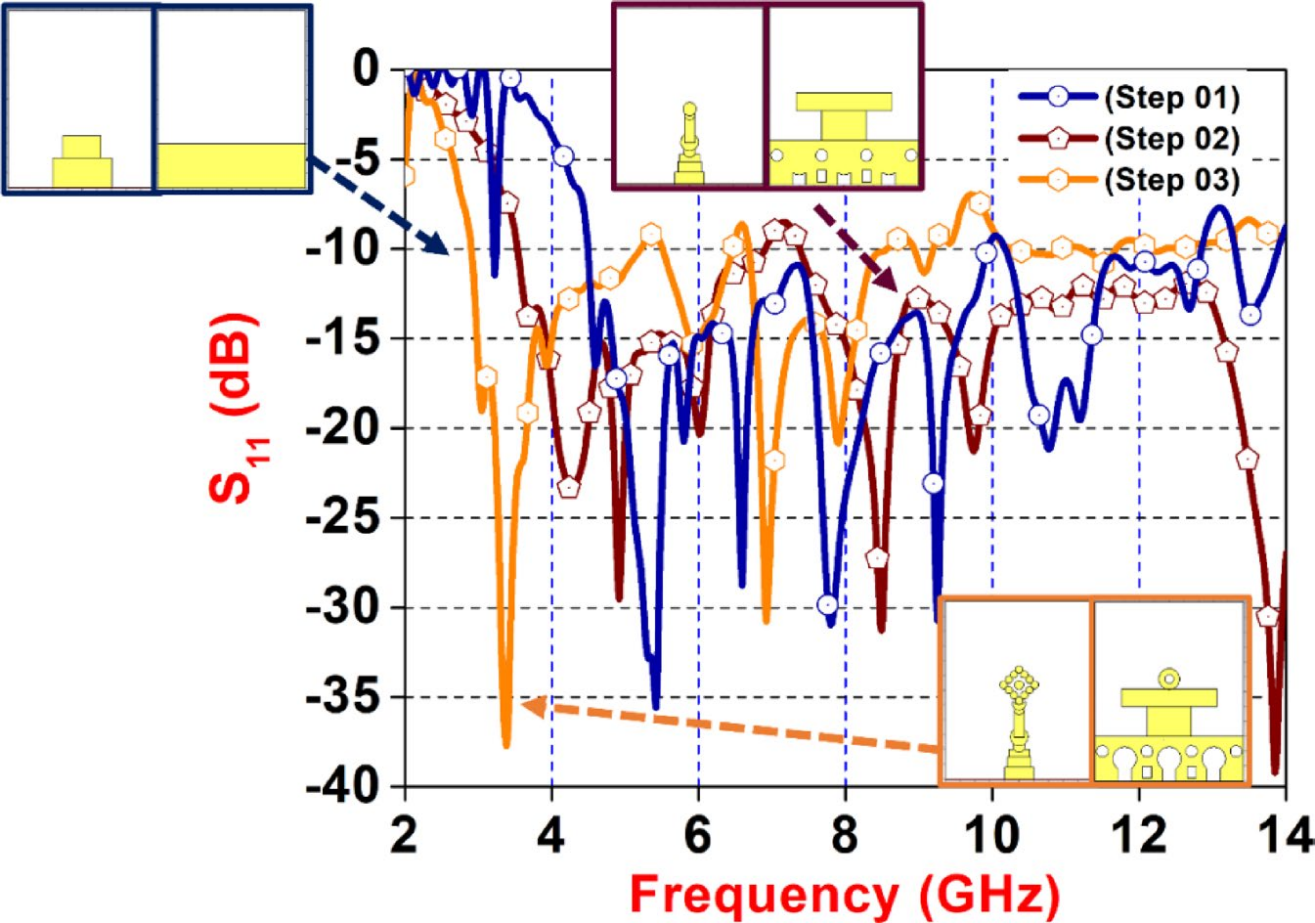
Reflection coefficient from step-01 to step-03.

Figure 4 presents a comparative analysis of the reflection coefficient (S₁₁) in dB versus frequency (GHz) for three evolutionary design stages of a planar UWB antenna: Step 01 (blue), Step 02 (maroon), and Step 03 (orange). The S₁₁ parameter, which indicates how much power is reflected back from the antenna, is plotted from 2 GHz to 14 GHz, where values below − 10 dB indicate good impedance matching and acceptable antenna performance. Step 01 shows moderate bandwidth and multiple resonances from 4.5 to 10 GHz, while Step 02 introduces additional design modifications, enhancing the number of resonance dips and dual frequency coverage from 3.8 to 6.8 GHz and 7.2 to 14 GHz. Step 03 further optimizes the structure, achieving ultra-wideband performance with deep and broad notches, especially visible around 4 GHz, 6 GHz, and 10 GHz.

**Fig. 5:**
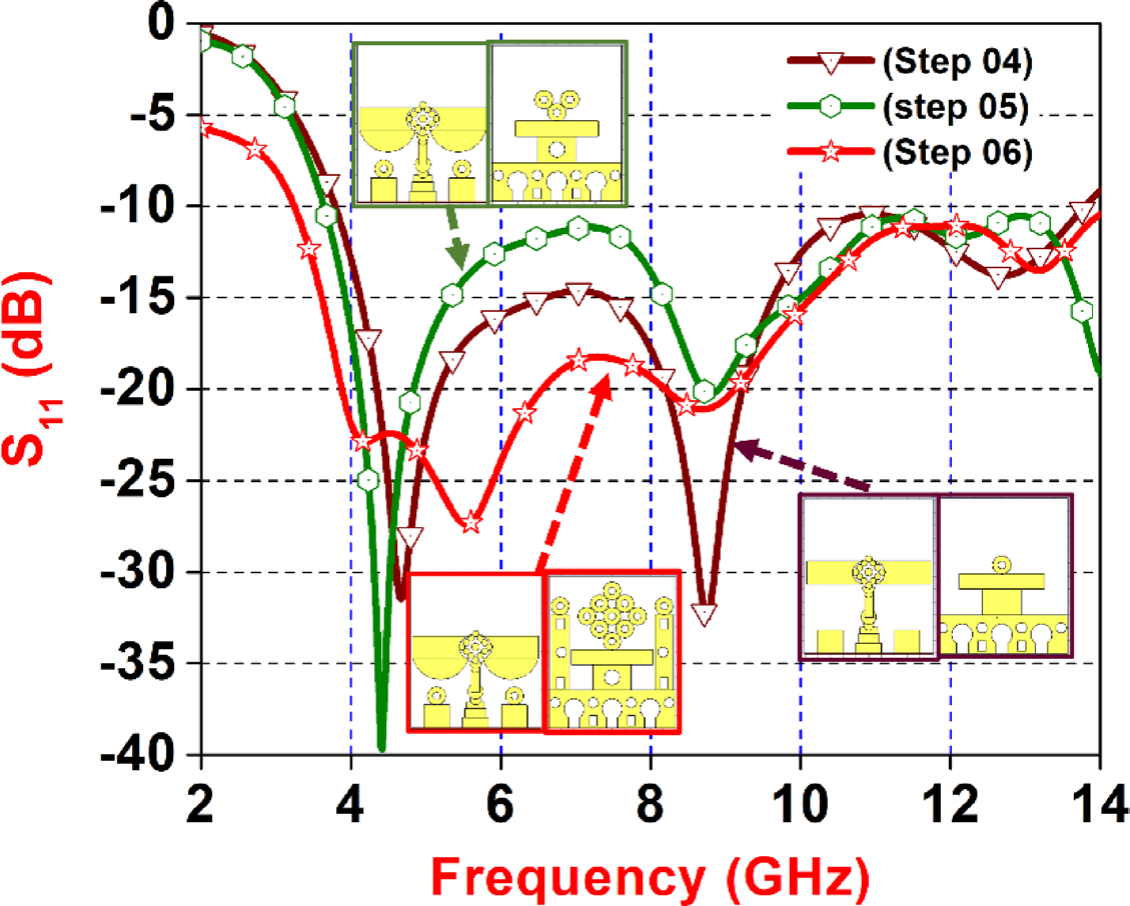
Reflection coefficient from step-04 to step-06.

Figure 5 displays a comparative plot of the reflection coefficient S_11_ in dB versus frequency (GHz) for the next three advanced design iterations of a planar UWB antenna, Step 04 (maroon, downward triangles), Step 05 (green, hexagons), and Step 06 (red, stars). Step 04 introduces new structural elements that lead to multiple resonance notches, particularly around 4.5 GHz and 13.7 GHz. Step 05 modifies the geometry further, which enhances bandwidth but slightly reduces notch depth from 3.9 to 14 GHz. The final configuration, Step 06, achieves an optimized design that combines wideband performance with distinct and deeper notches around 4.8 GHz and 8 GHz, along with a flatter response in the 10–14 GHz region and operates over a frequency range of 3.4 GHz to 14 GHz. Insets illustrate the geometric evolution at each step, where intricate etching patterns and symmetrical slot structures enhance the antenna’s rejection bands and frequency tuning capabilities. Overall, the progression from Step 04 to Step 06 reflects systematic structural enhancements that result in improved impedance bandwidth, targeted frequency notching. The Fig. 5 highlight the corresponding structural evolution of the antenna across the steps, emphasizing how geometrical modifications significantly improve frequency response.

**Fig. 6:**
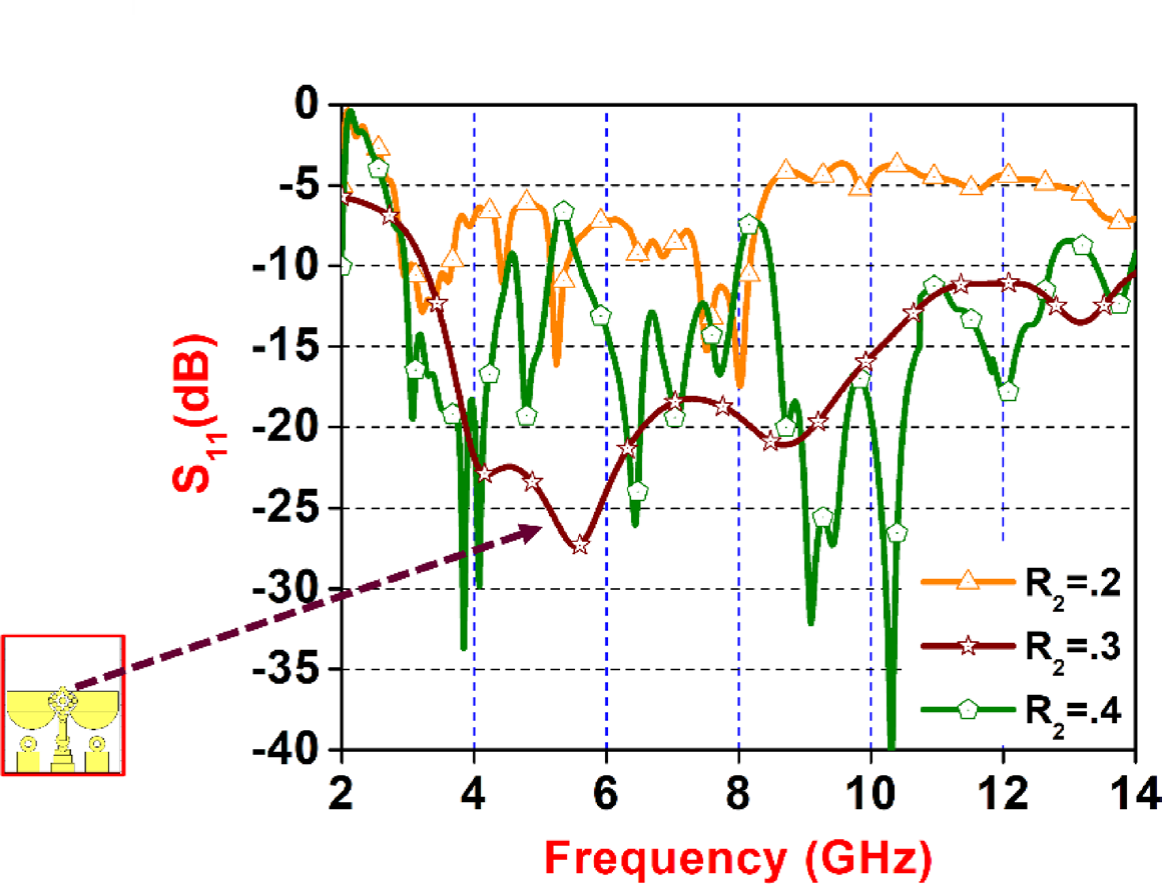
Parameter Sweep of ‘R_2_’. Variation.

Figure 6 illustrates the simulated return loss (S₁₁) against frequency for the proposed planar antenna, emphasizing the effect of altering the design variable R_2_​. The inset provides a detailed view of the antenna’s structure, including labelled dimensions and elements. The graph compares the antenna’s S₁₁ performance for three distinct values of R_2_​: 0.2, 0.3, and 0.4. When R_2_ = 0.2 (orange curve), the antenna shows weak resonance characteristics and higher return loss, indicating suboptimal impedance matching. With R_2_ = 0.3 (brown curve with stars), there is a significant improvement, with broader bandwidth and deeper notches observable between 3.4 GHz and 14 GHz. However, at R_2_ = 0.4 (green curve with pentagons), the antenna experiences impedance mismatch again. This figure clearly highlights how modifying a single structural parameter can have a notable impact on the antenna’s operational bandwidth and resonance behavior.

**Fig. 7:**
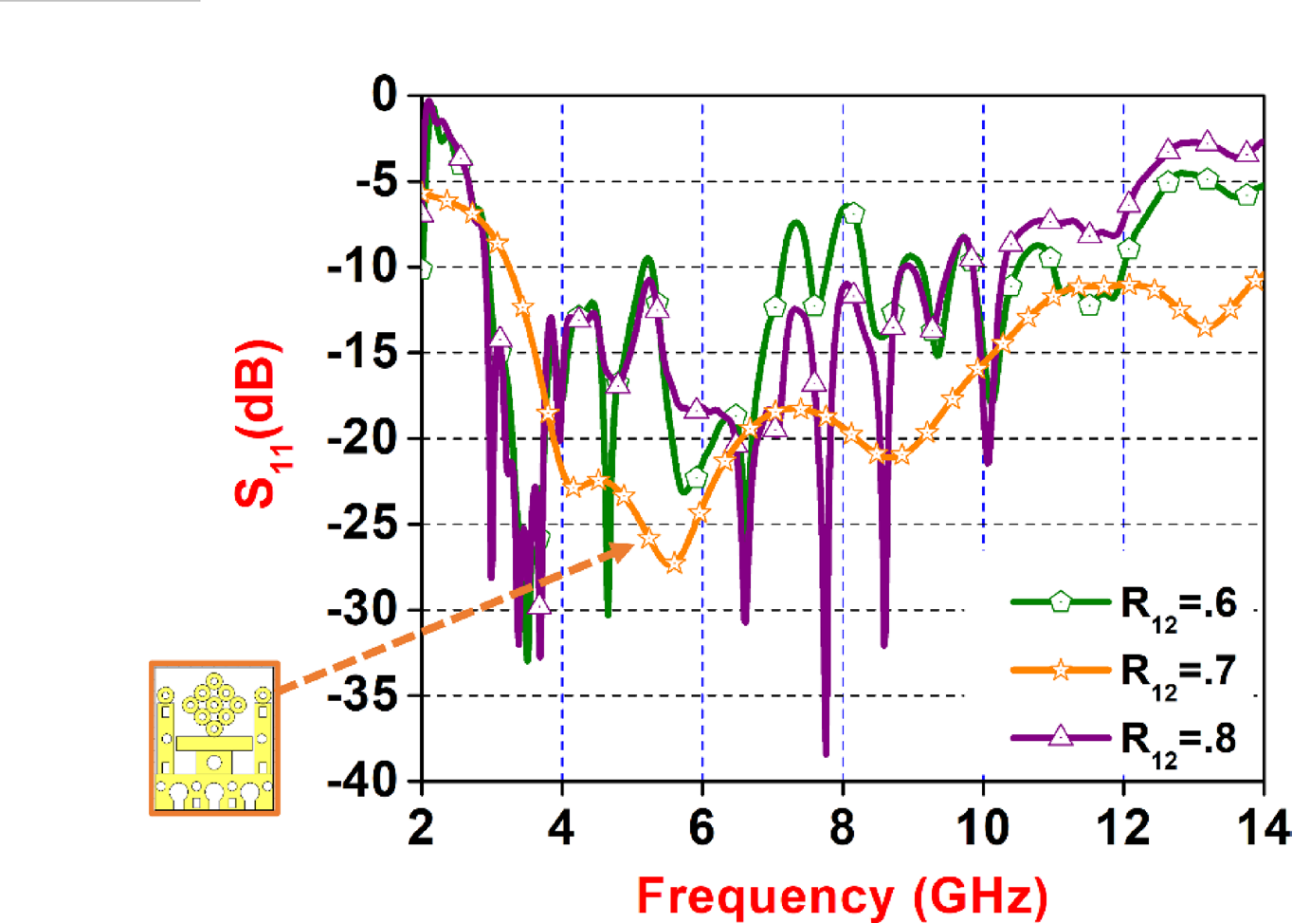
Parameter Sweep of ‘R_12_’. Variation.

Figure 7 illustrates the simulated return loss (S₁₁) versus frequency response of the proposed planar UWB antenna for varying values of the design parameter R_12_, specifically 0.6, 0.7, and 0.8. The inset provides a detailed view of the antenna’s structure, highlighting its geometrical features and dimensions. The S₁₁ curves demonstrate how changes in R_12_ influence the impedance matching and resonance behavior over the 2–14 GHz frequency band. Among the three variations, R_12_= 0.7 (represented by the orange curve) yields the most optimized performance, showing a well-matched response with broad impedance bandwidth from 3.4 to 14 GHZ and multiple sharp notches.

In contrast, R_12_= 0.6 (green) and R_12_= 0.8 (purple) exhibit relatively higher S₁₁ levels in certain bands, suggesting less effective matching. This comparison emphasizes the critical role of tuning R_12_ in achieving optimal antenna performance across the UWB spectrum.

**Fig. 8:**
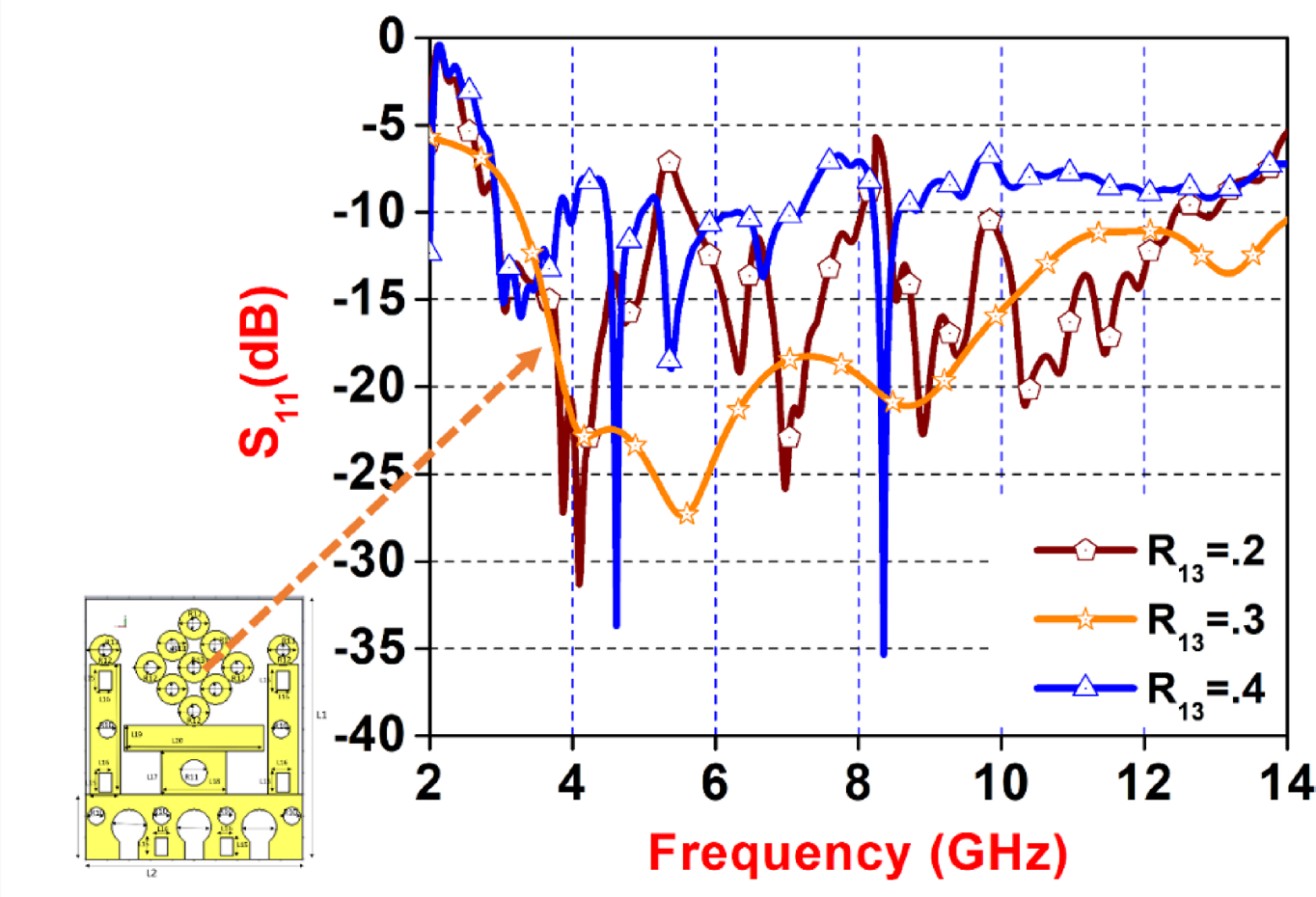
Parameter Sweep of ‘R_13_’. Variation.

Figure 8 presents a plot of the reflection coefficient (S₁₁ in dB) versus frequency (in GHz) for a planar UWB antenna, with performance compared for three different values of a geometrical parameter, R_13_, set at 0.2, 0.3, and 0.4. The frequency response ranges from 2 GHz to 14 GHz, illustrating the antenna’s impedance matching across the UWB spectrum. The brown curve (with pentagon markers) for R_13_=0.2 shows multiple deep notches below − 10 dB, indicating impedance mismatching at several frequencies. The orange curve (with star markers) for R_13_=0.3 exhibits a smoother response with proper impedance matching from 3.4 to 10 GHz, whereas the blue curve (with triangle markers) for R_13_=0.4 shows less variation and poorer matching at most frequencies. The inset at the bottom left corner displays the top view of the antenna structure with labelled circular slots, suggesting that R_13_ is an optimum design parameter influencing the slot dimensions.

## Results and discussion

**Fig. 9:**
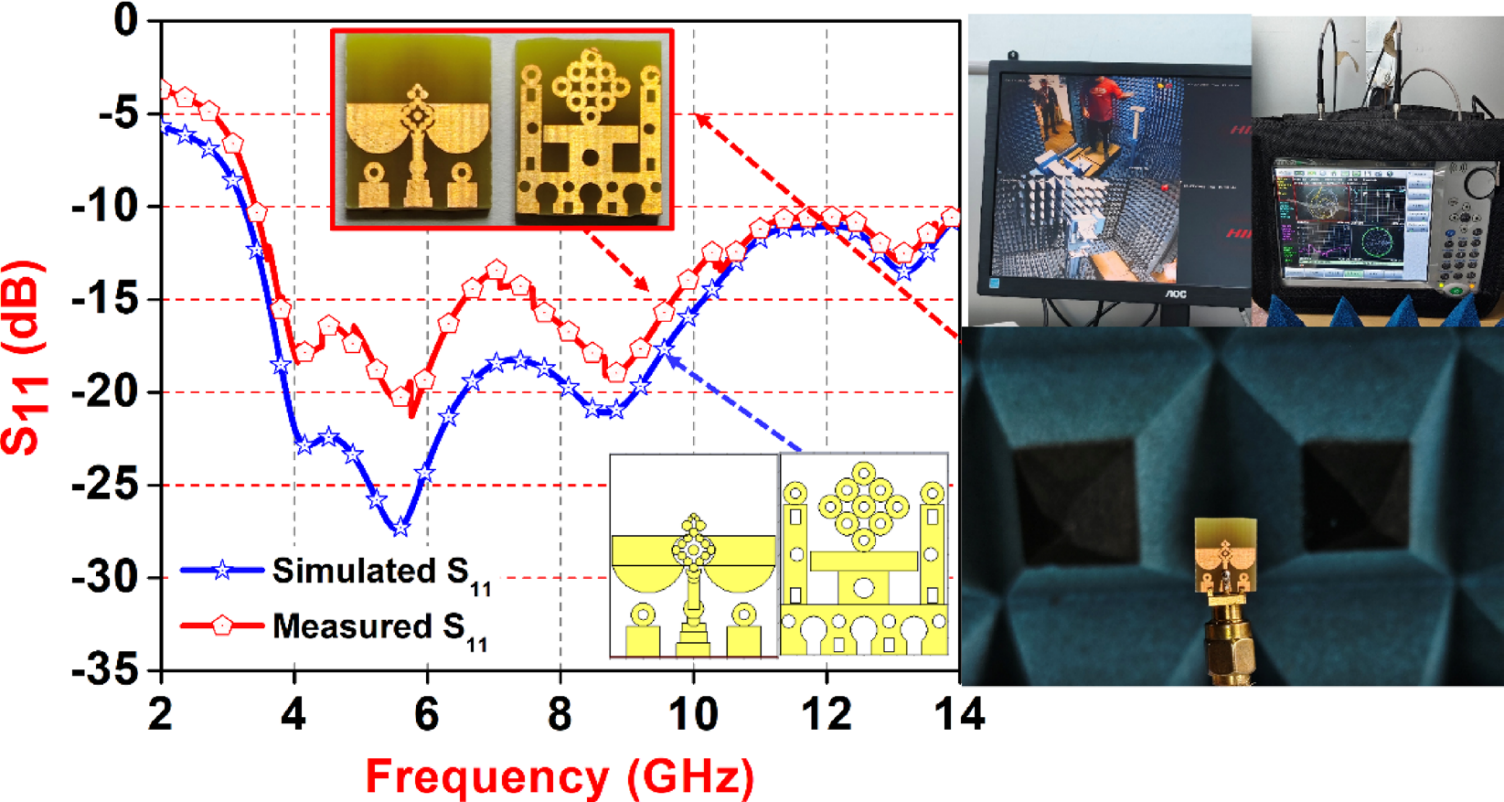
Comparison of reflection coefficient (S_11_).

Figure 9 comparison between simulated and measured S₁₁ (return loss) characteristics of the proposed UWB antenna across the 2–14 GHz frequency range and its resonating from 3.4 to 14 GHZ. The simulated results (blue curve with star markers) demonstrate strong impedance matching with return loss levels below − 30 dB around 6 GHz, while the measured results (red curve with pentagon markers) closely follow the simulated trend, with minor deviations attributed to fabrication and measurement uncertainties. The proposed antenna was fabricated in the Antenna Laboratory, and performance evaluation was carried out in an anechoic chamber to ensure accurate, interference-free results. A Vector Network Analyzer (VNA) was used to measure return loss, impedance, and radiation pattern. The antenna was precisely positioned, and measurements were repeated at different angles to assess directivity and confirm consistent behavior. Insets show the fabricated antenna prototype and its simulated layout, verifying successful realization and validation of the design.

**Fig. 10:**
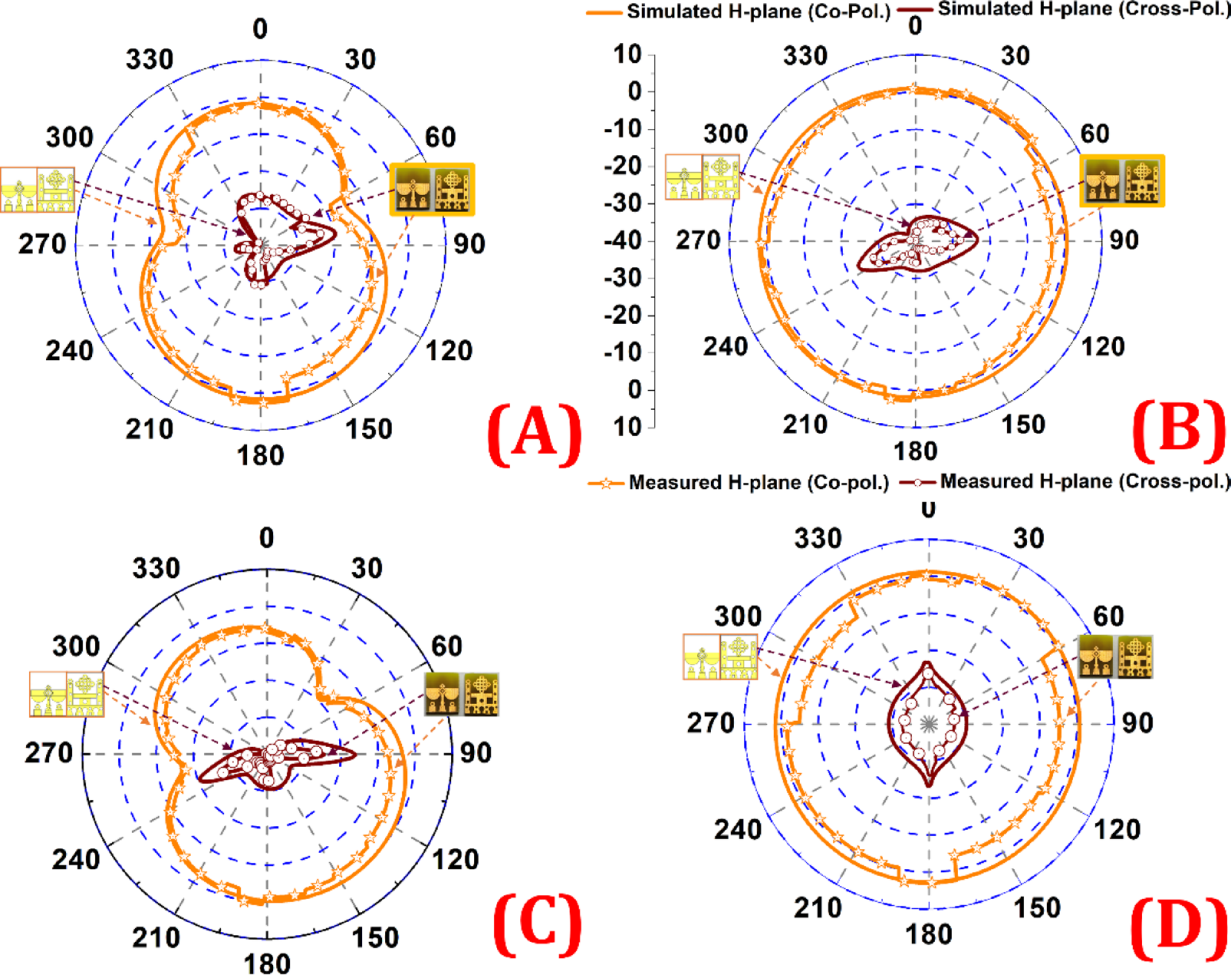
Simulated and measured E-plane and H-plane patterns at 5 GHz and 8 GHz, showing dominant co-polarization and cross-polarization levels below − 20 dB, indicating stable and pure radiation performance.

Figures 10A, B illustrate the simulated E-plane and H-plane radiation characteristics of the proposed antenna at 5 GHz. The co-polarized component exhibits a nearly omnidirectional radiation pattern with good symmetry, while the cross-polarized levels remain significantly lower, consistently below − 20 dB relative to the co-polarization, demonstrating strong polarization purity. The measured results closely follow the simulated curves, with minor variations attributed to fabrication tolerances and measurement setup.

Figures (C) and (D) present the antenna performance at 8 GHz. The co-polarized field continues to dominate the radiation response, while the cross-polarized field remains well suppressed, again below − 20 dB across most angular regions. The measured patterns align well with the simulations, confirming stable and reliable antenna behavior at higher frequencies.

Overall, the antenna demonstrates balanced and consistent radiation profiles in both E- and H-planes at 5 GHz and 8 GHz, with low cross-polarization and dominant co-polarization. This confirms its suitability for wideband and multi-band communication applications.

In practical CubeSat missions, antennas are commonly mounted on metallic satellite panels or operate in close proximity to conductive housings, which can influence impedance matching, radiation efficiency, and radiation characteristics due to surface current coupling and altered boundary conditions. The proposed antenna has been intentionally designed with a compact electrical size (0.113λ × 0.136λ × 0.017λ at 3.4 GHz) and a defected ground structure (DGS), which helps suppress excessive surface currents and reduces sensitivity to nearby metallic structures. When integrated onto a CubeSat platform or metallic enclosure, minor resonance shifts and slight efficiency degradation may occur; however, owing to the ultra-wide impedance bandwidth of 3.4–14 GHz (121.8%), the antenna maintains |S₁₁| below − 10 dB across the operational band. Moreover, the quasi-omnidirectional radiation patterns observed at 5 GHz and 8 GHz remain stable, making the antenna suitable for CubeSat missions where satellite orientation continuously varies. The planar, low-profile configuration also enables flush mounting on CubeSat faces, minimizing mechanical obstruction and electromagnetic interference with onboard subsystems. These characteristics confirm the robustness of the proposed antenna for CubeSat platform integration and metallic housing environments.

### Mathematical calculations

Wavelength (λ) Calculation

The wavelength is given by

$$\:\boldsymbol{\lambda\:}=\frac{\boldsymbol{c}}{\boldsymbol{f}}$$.

where $\:c=3\times\:{10}^{8}$m/s is the speed of light.

At $$\:\boldsymbol{f}=3.4\:$$GHz:

1 $$\lambda _{{{\mathrm{max}}}} = \frac{{3 \times 10^{8} }}{{3.4 \times 10^{9} }} = 88.2\;{\mathrm{mm}}$$

At $$\:\boldsymbol{f}=14\:$$GHz:

2 $$\lambda _{{{\mathrm{min}}}} = \frac{{3 \times 10^{8} }}{{14 \times 10^{9} }} = 21.4\;{\mathrm{mm}}$$

### Electrical size calculation

The electrical size is defined as the ratio of the physical dimension to the operating wavelength.

At 3.4 GHz:

3 $${\mathrm{Electrical}}\;{\mathrm{Size}} = \frac{{10\;{\mathrm{mm}}}}{{88.2\;{\mathrm{mm}}}} \approx 0.113\lambda$$

At 14 GHz:

4 $${\mathrm{Electrical}}\;{\mathrm{Size}} = \frac{{10\;{\mathrm{mm}}}}{{21.4\;{\mathrm{mm}}}} \approx 0.467\lambda$$

Hence, the electrical dimensions of the structure are approximately.

$$\:0.113\boldsymbol{\lambda\:}\times\:0.136\boldsymbol{\lambda\:}\times\:0.017\boldsymbol{\lambda\:}$$

### Impedance bandwidth calculation

The fractional impedance bandwidth is calculated using:

5 $${\mathrm{BW}}\left( {{\% }} \right) = \left( {\frac{{f_{{{\mathrm{high}}}} - f_{{{\mathrm{low}}}} }}{{f_{{{\mathrm{center}}}} }}} \right) \times 100$$

where

- $$\:{\boldsymbol{f}}_{\mathrm{low}}=3.4$$GHz
- $$\:{\boldsymbol{f}}_{\mathrm{high}}=14$$GHz

$$\:{f}_{\mathrm{center}}=\frac{{f}_{\mathrm{low}}+{f}_{\mathrm{high}}}{2}=\frac{3.4+14}{2}=8.7\:{\mathrm{GHz}}.$$

Thus,

6 $${\mathrm{BW}}\left( {{\% }} \right) = \left( {\frac{{14 - 3.4}}{{8.7}}} \right) \times 100 = 121.8{{\% }}$$

**Fig. 11:**
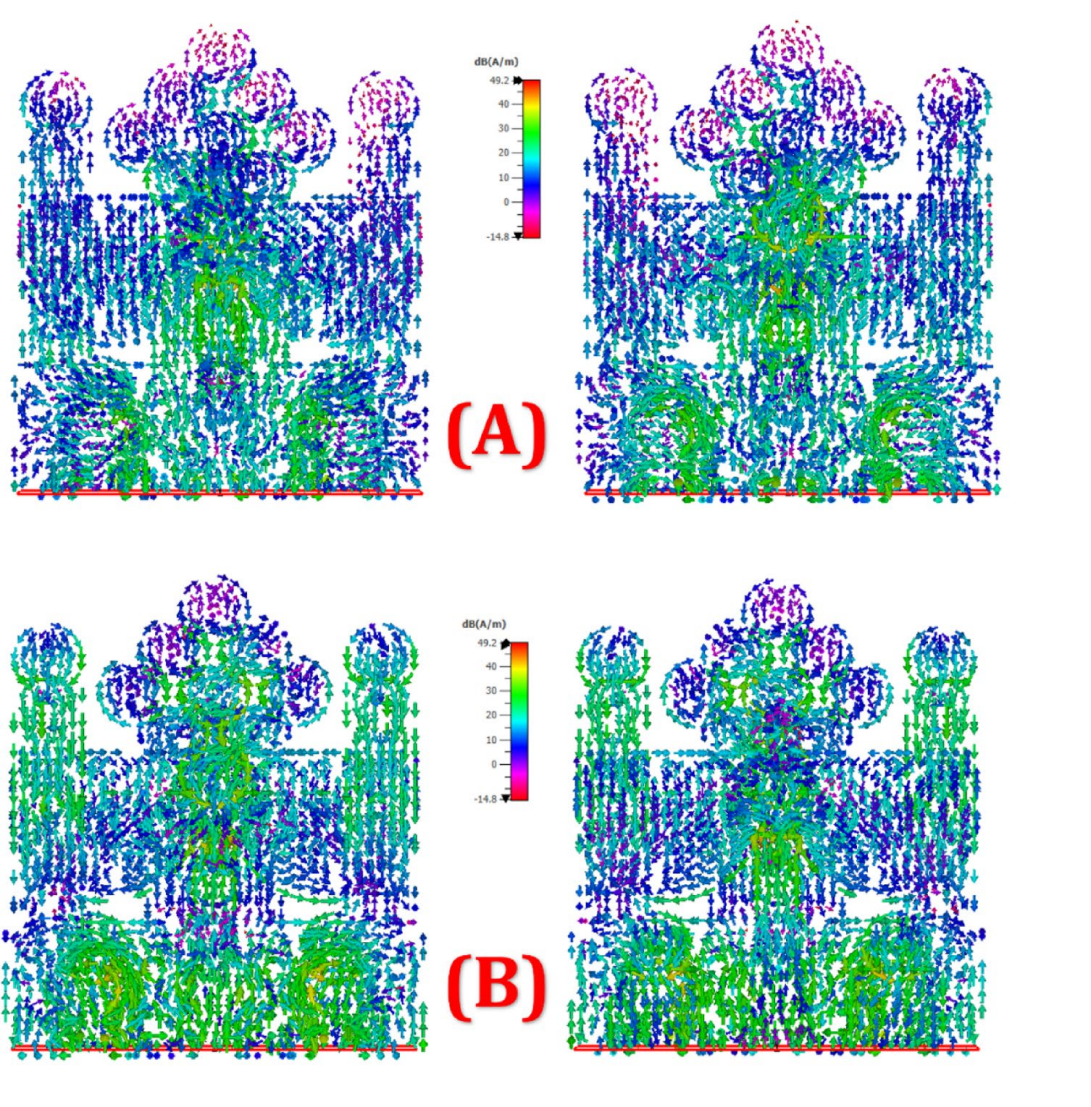
Simulated surface current distribution at (**A**) 5 GHz front and back and (**B**) 8 GHz front and back.

Figure 11 presents surface current distribution plots at either 5–8 GHz for two configurations labelled (A) and (B). Each configuration contains two views (likely front and back or different polarizations), illustrating the electromagnetic surface current intensity on a symmetrical, ornate structure, possibly an antenna or metamaterial design. The colour scale, measured in dB(A/m), ranges from approximately − 14.8 to + 49.2 dB, with colours transitioning from purple (low current) through blue and green to red (high current). In both configurations, strong currents (yellow to red) concentrate primarily near the central and lower parts of the structure, suggesting regions of intense electromagnetic activity. Configuration (A) shows more symmetrical and vertically aligned current paths, while (B) appears to exhibit slightly more dispersed or shifted current patterns, possibly due to geometrical or feed variation. These visualizations help in understanding how surface currents behave at high-frequency excitation, essential for optimizing radiating performance or suppressing unwanted emissions.

**Fig. 12:**
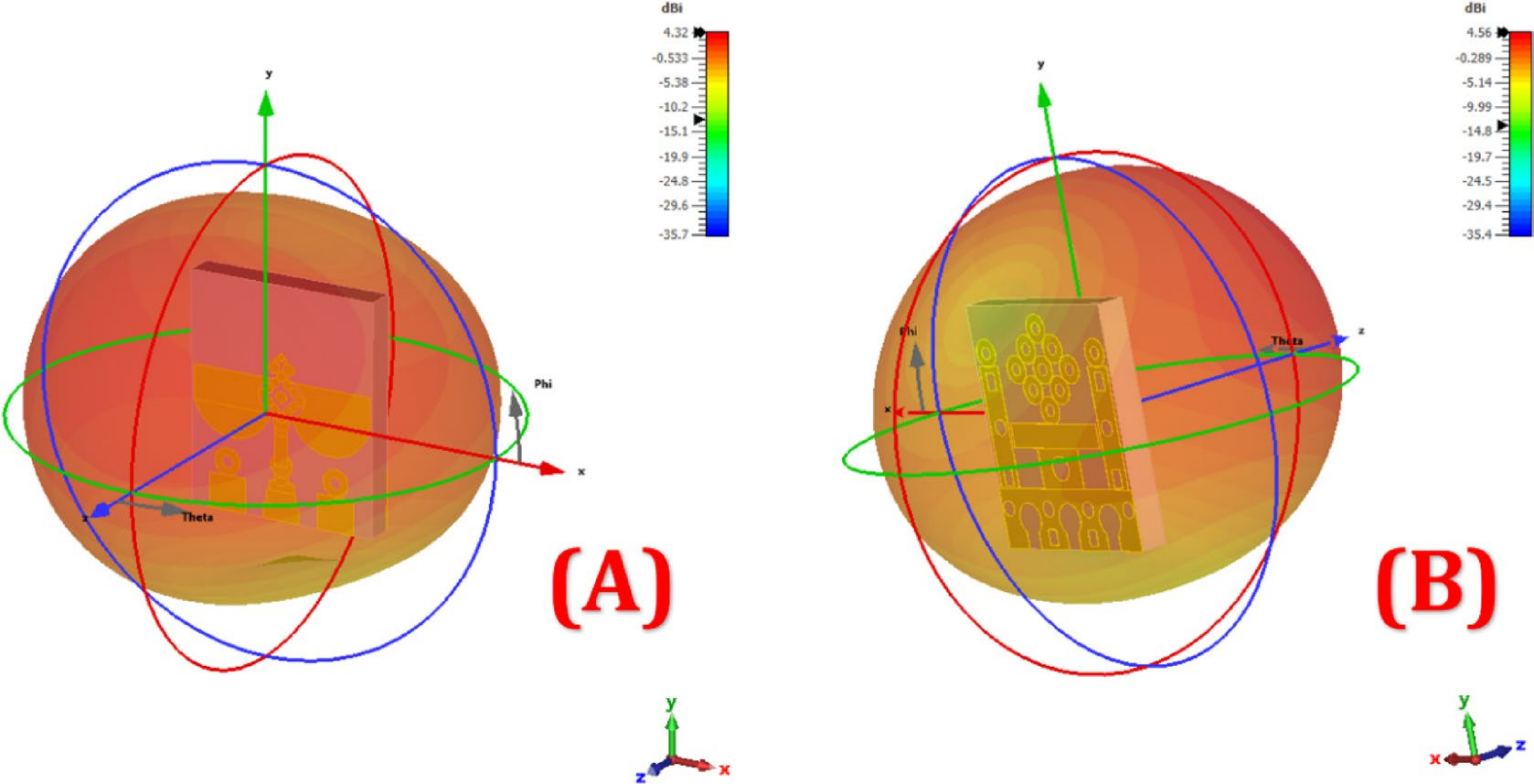
Radiation antenna lobe patterns (**A**) left phase lobe’ at 8 GHz and (**B**) right phase lobe’ at 5 GHz.

Figure 12 illustrates the 3D radiation patterns of two antenna configurations labelled (A) and (B), highlighting the spatial distribution of radiated energy in dBi. The radiation lobes are depicted using coloured volumetric plots, where red and orange areas represent stronger radiation intensity, and blue indicates weaker emission levels. Configuration (A) exhibits an omnidirectional pattern with slightly enhanced radiation along the y-axis, suggesting broad and uniform coverage suitable for wide-area communication. In contrast, Configuration (B) shows a more directional pattern, with a focused main lobe and slightly reduced back radiation, indicating better directivity. The maximum gains are approximately 4.32 dBi for (A) and 4.56 dBi for (B), as indicated by their respective colour bars. Both designs maintain smooth and stable radiation characteristics with well-distributed theta and phi cuts, which are crucial for ensuring consistent performance in real-world wireless systems. These patterns confirm that the antennas are capable of efficient three-dimensional radiation with minimal distortion or nulls.

**Fig. 13:**
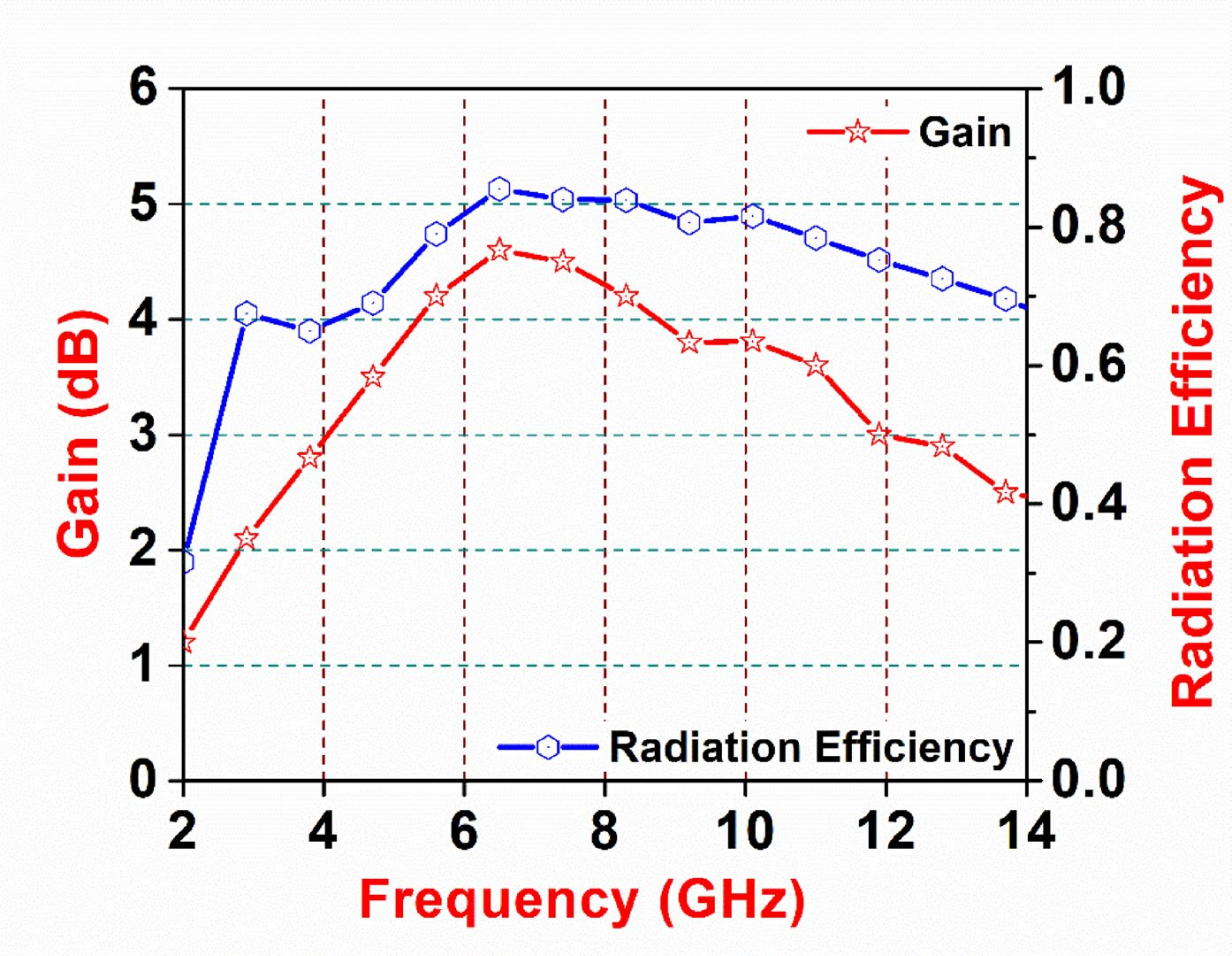
Gain and radiation efficiency variation versus frequency for the proposed antenna.

Figure 13 illustrates the variation of antenna gain and radiation efficiency as a function of frequency over the 2–14 GHz range. It can be observed that the gain gradually increases from the lower frequencies, reaching a maximum value of about 4.5–4.6 dBi around 6–7 GHz, and then shows a smooth, controlled reduction toward the upper end of the band while remaining above approximately 2.5 dBi. This trend indicates stable radiating behavior without abrupt degradation across the ultra-wide operating band.

In parallel, the radiation efficiency remains consistently high throughout the frequency range, exceeding 70% over most of the band and attaining a peak efficiency of nearly 83% in the mid-band region. Even at higher frequencies, the efficiency shows only a gradual decline, demonstrating effective power radiation and limited losses. Overall, the combined gain and efficiency response confirms that the proposed antenna maintains reliable and stable radiation performance across its entire operating bandwidth, supporting its suitability for wideband communication applications.

The comparison Table 2 emphasizes the distinct performance differences between conventional antenna designs and proposed antennas across several key parameters.

**Table 2:** Comparison table.

Ref.	$\:\left(\boldsymbol{\eta\:}\right)$ (%)	Gain (dBi)	Band (GHz)	B/W (%)	Electrical size (in λ)
^2^	NA	3.6	26–32	19.89%	.05λ*.07λ*.013λ
^3^	78%	4.9	38–54	34%	.076λ*.083λ*.019λ
^4^	89%	5.1	3.1–11	110%	.20λ*.25λ*.015λ
^5^	80%	2.6	28–50	56%	.10λ*.078λ*.015λ
^6^	NA	3.56	25.3–26.8	< 1%	.05λ*.05λ*.012λ
^7^	62%	4.5	2–9	127%	0.33 λ*.22λ*0.1 λ
^10^	81%	3.2	3.5–19	145%	.23λ*.23λ*.015λ
^11^	NA	4.4	9.5–10.36.5.36	< 1%	.6λ*.06λ*.004λ
^13^	87%	5.2	2.9–16	139%	.33λ*.24λ*.014λ
^14^	72%	2.79	2.8–12	122%	.18*λ* *.14*λ* *.15*λ*
^15^	78.3%	2.3	2.7–7.3	108%	.32λ*.2λ*.014λ
^12^	NA	5	2.84–5.17	58%	.84λ*.68λ*.06λ
^16^	NA	4.3	42.48–45.2	87%	.10λ*.12λ*.02λ
^21^	NA	5.48	57.9–62	68%	.15λ*.15λ*.02λ
^27^	NA	2.52	2.9–4.48	42.8%	.02λ*.023λ*.001λ
Presented	82.9%	4.56	3.4–14	121.8%	.11λ*.13λ*.017λ

## Conclusion

A compact wideband antenna with dimensions of 10 × 12 × 1.5 mm³ has been successfully designed, fabricated, and tested on an FR4 substrate. The antenna achieves an extensive operating range from 3.4 to 14 GHz, delivering an impedance bandwidth of 121.8%. Experimental results confirm stable radiation patterns, high efficiency of 82.9%, a return loss of −28 dB, and a peak gain of 4.56 dBi, aligning well with simulation outcomes. The analysis of surface currents and radiation behavior further validates its effectiveness across multiple frequency bands. Owing to its wide coverage, compact size, and robust performance, the proposed design is well-suited for applications in S-band, C-band, X-band, 5G communication, defense systems, and CubeSat platforms.

## Data Availability

No new data were generated during the study. All the data are contained within the manuscript.
